# University of Buffalo

**Published:** 1849-11

**Authors:** 


*University of Buffalo.* — The preliminary term of instruction in the Medical Department of the University of Buffalo, commenced on the 10th ult. and is now in progress. Fifty-five students are already in attendance at this term, and the prospect is, that the class for the regular session, which commences on the 7th inst., will be large. Lectures are daily given in the new college edifice; clinical attendance at the Hospital, Wednesdays and Saturdays; the remainder of the time being occupied with anatomical demonstrations, and dissections. The regular session will be opened by a public introductory discourse, and other exercises, to which members of the Profession in the vicinity are respectfully and cordially invited. We reserve some account of the new edifice, as completed, for another number.

